# Identification of serum miR-378 and miR-575 as diagnostic indicators and predicting surgical prognosis in human epilepsy

**DOI:** 10.5937/jomb0-32988

**Published:** 2022-04-08

**Authors:** Xiuxiu Li, Zhiqing Gao, Mei Ling Ma, Li Li, Shifeng Guo

**Affiliations:** 1 Linyi Central Hospital, Department of Neurology, Linyi City, Shandong Province, China

**Keywords:** miR-378, miR-575, human epilepsy, serum biomarkers, miR-378, miR-575, epilepsija kod ljudi, serumski biomarkeri

## Abstract

**Background:**

Epilepsy (EP) is a common neurological disorder which is characterized by excessive abnormal synchronization of neuronal discharges in the brain due to chronic recurrent seizures of multiple etiologies. Variety of microRNAs have been associated with the occurrence and development of EP. This study aimed to determine the aberrant expression of miR-378 and miR-575 in EP patients to validate their potential to distinguish EP from healthy patients.

**Methods:**

RT-qPCR was used to determine the expressions of miR-378 and miR-575 from serum specimens of 106 EP and 103 control individuals. Clinical indicators between EP patients and controls were assessed. Based on surgical outcome, EP patients were further divided into Engel I-IV EP. The potentials of miR-378 and miR-575 in discriminating EP from healthy participants and predicting surgical prognosis were calculated by receiver operating characteristic (ROC) analysis.

**Results:**

We found the miR-378 and miR-575 were significantly declined (P<0.001) in Engel I-II and III-IV EP patients with no difference in clinical parameters compared. Moreover, miR-378 and miR-575 displayed high sensitivity, specificity, and accuracy in distinguishing EP patients and predicting surgical outcomes. Moreover, after surgical treatment, miR-378 and miR-575 levels were increased compared with those at admission, suggesting their potentials in treatment response.

**Conclusions:**

miR-378 and miR-575 could be utilized as novel and non-invasive serum biomarkers in discriminating EP from healthy controls and predicting surgical outcome, shedding new insights on epileptogenesis and EP treatment.

## Abstract (sr)

**Uvod:**

Epilepsija (EP) je uobičajen neurološki poremećaj koji se karakteriše prekomernom sinhronizacijom pražnjenja neurona u mozgu usled hroničnih ponovljenih napada različitih etiologija. Različite mikro RNK su povezane sa pojavom i razvojem EP. Ova studija je imala za cilj da odredi aberantnu ekspresiju miR-378 i miR-575 kod pacijenata sa EP kako bi potvrdila njihov potencijal da se ustanovi razlika izme|u pacijenata sa EP i zdravih pacijenata.

**Metode:**

RT-qPCR je korišćen da bi se odredila ekspresija miR-378 i miR-575 iz serumskih uzoraka 106 pacijenata sa EP i 103 kontrolna ispitanika. Izvršena je procena kliničkih pokazatelja kod pacijenata sa EP i kontrolnih ispitanika. Na osnovu ishoda hirurškog lečenja, pacijenti sa EP su dalje podeljeni na Engel I-IV EP. Analizom krive operativnih karakteristika (ROC) izračunat je potencijal koji imaju miR-378 i miR-575 i koji karakteriše pacijente sa EP u odnosu na zdrave učesnike istraživanja kako bi se predvideo tok lečenja.

**Rezultati:**

Utvrdili smo da su miR-378 i miR-575 značajno smanjeni (P < 0,001) kod pacijenata sa Engel I-II i III-IV EP bez razlika u upore|enim kliničkim parametrima. Štaviše, miR-378 i miR-575 su pokazali visoku osetljivost, specifičnost i tačnost u razlikovanju EP pacijenata i predvi |anju ishoda. Dalje, nakon hirurškog lečenja, nivoi miR-378 i miR-575 su povećani u pore|enju sa onima pri prijemu, što ukazuje na njihov potencijal u rekaciji na lečenje.

**Zaključak:**

miR-378 i miR-575 se mogu koristiti kao novi i neinvazivni biomarkeri u serumu za diferencijaciju pacijenata sa EP od zdravih kontrolnih ispitanika i predvi|anje hirurškog ishoda, otkrivajući nove uvide u epileptogenezu i lečenje EP.

## Introduction

Epilepsy (EP) is a chronic brain disease with a long course that poses a severe threat to patients' physical and mental health [1]. Seizures caused abnormal synchronous neuronal discharges [2], resulting in transient brain dysfunction and abnormalities in neuronal apoptosis, necrosis, and neurological deficits [3]. In addition, EP was characterized by transience, unpredictability, and recurrence [4]. About half of epilepsy patients had their onset in childhood and teenage years [5]. So far, magnetic resonance imaging (MRI) and electroencephalography (EEG) have been able to help diagnose and differentiate epilepsy, whereas the assessment of anti-epileptic efficacy depended on EEG [6]. However, EEG is expensive and time-consuming, which is not suitable for frequent and quick applications [7]. Therefore, it is imperative to explore non-invasive and economic biomarkers to monitor EP development and evaluate the effectiveness of anti-epileptic treatment.

MicroRNAs (miRNAs) are endogenous singlestranded small RNA with approximately 22–24 nucleotides in length [8]. These miRNAs are highly conserved and play a post-transcriptional role in regulating gene expressions [9]. Several miRNAs are released from cells and circulate in the blood, show resistance to various RNA digesting enzymes, and are not affected by storage time, pH, temperature, repeat freezing, and thawing [10]. Because these are less affected by changes in the peripheral environment, miRNAs are used as biomarkers for various diseases, including epilepsy [11] [12]. In recent years, there has been increasing evidence indicating that multiple miRNAs regulate EP. For instance, the expression of miR-106b-5p, let-7d-5p, miR-130a-3p and miR-146a-5p are upregulated in the serum of EP patients, while miR-15a-5p and miR-194-5p levels are downregulated [13]. Moreover, miR-134 and miR-21-5p are downregulated among epileptic rats [14] [15]. Meanwhile, multiple miRNAs have been associated with epileptogenesis and the progression of EP [16] [17]. In a previous study, miR-378 and miR-575 are dysregulated during the onset of EP and post-treatment [18]. However, the potential of miR-378 and miR-575 concerning EP diagnosis and outcome prediction remained unknown.

In the present study, we aimed to determine the aberrant expression of miR-378 and miR-575 in EP patients to determine the potential in differentiating EP from healthy individuals. Moreover, the dynamic changes of miR-378 and miR-575 during seizure onset and after surgical treatment were evaluated to validate their application in predicting EP surgical outcome.

## Materials and Methods

### Samples collection

A total of 106 individuals diagnosed with EP but remained untreated in Linyi Central Hospital were enrolled as the EP group from November 2017 to April 2020. The control group consisted of 103 healthy individuals who underwent a physical examination at our hospital during the same period. The EP group and the healthy group were age and sexmatched. All enrolled participants signed informed consent, and the Ethics Committee of Linyi Central Hospital approved the current study protocol. Inclusion criteria for the EP: (1) those who were eligible for primary EP according to the diagnostic and classification criteria of the International League Against Epilepsy (ILAE); (2) the time since the last seizure was less than 1 week; (3) organic lesions such as traumatic brain injury, developmental malformation of the brain, intracranial occupancy, and cerebral infarction were excluded by imaging examinations such as computed tomography (CT), MRI and EEG. In addition, EP patients with neurological and other systemic disorders, traumatic brain injury, or receiving psychotropic medications in the last three months were excluded.

5 mL of blood sample was collected from EP patients and healthy controls after fasting and kept at room temperature for 3 hours. Then, the supernatant was aspirated into a sterilized EP tube. The sample was then centrifuged at 3000 r/min at room temperature for 10 min. After centrifugation, samples were stored at -80°C until use.

### RT-qPCR analysis

The frozen serum samples, stored at-80°C, were thawed at 4°C in a refrigerator. 1.5 mL of each sample was dispensed in a centrifuge tube. Next, 1 mL TRIzol was added to each sample at room temperature for 5 min. After lysis, 200 mL of chloroform was added for 10 min and then centrifuged at 12,000 g for 15 min at room temperature. Afterwards, 1 mL of 75% alcohol was used to precipitate RNA and then centrifuged at 4°C for 5 min at 7,500 g. After washing, the RNA was placed for 15 min to dry RNA. Finally, 20 μL DNase/RNase-free deionized water was added to dissolve the RNA extract. The samples were analyzed for RNA purity using a NANODROP 2000 spectrophotometer. Then, the reverse transcription kit, SuperScript R Enzyme Mix, was used to prepare cDNA. The RT-qPCR was performed in AXYGEN 0.2 mL 96-well PCR system with U6 RNA as the internal reference. The CT values of miR-378 and miR-575 were derived at the end of the amplification reaction by fluorometric quantification and analyzed using the 2-∆∆CT method. All the experiments were performed in triplicate.

### Statistical analysis

The experimental data, for normal distribution and variance homogeneity, were expressed in the form of mean ± standard deviation (x̄±s). SPSS 22.0 software was used to statistically analyze the data obtained from each group, and an independent t-test and chi-square test were used for quantification. P<0.05 was deemed statistically significant.

## Results

### Clinicopathological information of all participants

As shown in Table 1, there were no significant differences in age, gender distribution, BMI, and smoking history between EP patients and healthy controls.

**Table 1:** Clinical information of healthy individuals and patients with epilepsy

Parameters	EP patients<br> (n = 106)	Healthy<br> (n = 103)	P value
Gender			
Male	57	52	0.6342
Female	49	51	
Age	29.5 ± 7.2	31.2 ± 6.8	0.0809
EP duration (years)			
≤ 5	81	/	/
> 5	25	/	
Smoking history			
Yes	44	46	0.6456
No	62	57	
BMI (kg/m^2^)	24.6 ± 0.7	25.0 ± 0.5	0.9555
miR-378 level	0.47 ± 0.17	1.01 ± 0.26	<0.0001
miR-575 level	0.49 ± 0.21	1.03 ± 0.24	<0.0001

### Down-regulated miR-378 and miR-575 expressions in serum samples in EP patients

**Figure 1:**
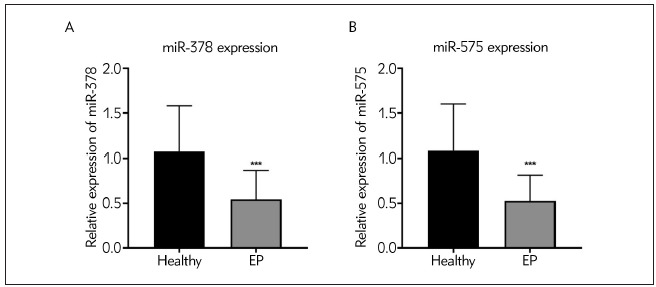
Aberrant expressions of miR-378 and miR-575 in serum samples from healthy controls and patients with EP (A) Expressions of miR-378 were evaluated by qRT-PCR analysis. ***P<0.001, EP vs Healthy. (B) Expressions of miR-575 were evaluated by qRT-PCR analysis. ***P<0.001, EP vs Healthy. EP, epilepsy

We used RT-qPCR to analyze and measure the expression levels of miR-378 and miR-575 in serum samples from EP patients and healthy volunteers. As highlighted in Figure 1A and Figure 1B, the expression of miR-378 and miR-575 were prominently increased in the EP group as compared with the healthy control (^***^P<0.001).

**Figure 2:**
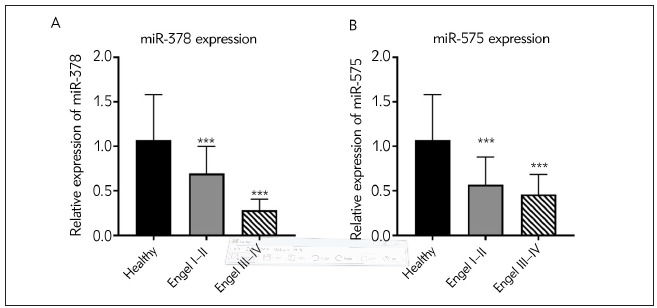
Different expressions of miR-378 and miR-575 in patients with EP according to Engel grades (A) miR-378 expressions in Engel I–II and Engel III–IV EP patients. (B) miR-575 expressions in Engel I–II and Engel III–IV EP patients.<br>***P<0.001, Engel I–II vs. Healthy, Engel III–IV vs. Healthy. EP, epilepsy

Engel grades were applied to discriminate EP patients regarding seizure response to surgical treatment. According to the guidelines, Engel I was defined as free of disabling EP; Engel II was rare seizure after surgery; Engel III was deemed worth-while improvement of EP reduction after surgery; Engel IV was characterized as no improvement in seizure conditions. As depicted in Figure 2A and Figure 2B, Engel III-IV EP patients had significantly lower miR-378 and miR-575 expressions than Engel I-II (^***^P<0.001), suggesting the potentials of miR-378 and miR-575 in predicting surgical outcome.

### Diagnostic performances of miR-378 and miR-575 in distinguishing EP patients from healthy controls

**Figure 3:**
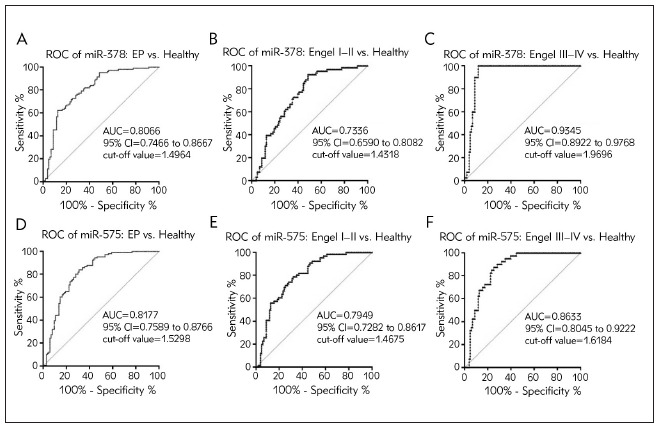
ROC analysis of miR-378 and miR-575 in discriminating EP patients from healthy controls (A) AUC of miR-378 in differentiating EP patients from healthy volunteers. (B) AUC of miR-378 in differentiating Engel I–II EP patients from healthy volunteers. (C) AUC of miR-378 in differentiating Engel III–IV EP patients from healthy volunteers. (D) AUC of miR-575 in differentiating EP patients from healthy volunteers. (E) AUC of miR-575 in differentiating Engel I–II EP patients from healthy volunteers. (F) AUC of miR-575 in differentiating Engel III–IV EP patients from healthy volunteers

ROC analysis was conducted to evaluate the capabilities of miR-378 and miR-575 abnormal expressions in discriminating EP from healthy controls. As demonstrated in Figure 3A and Figure 3D, miR-378 and miR-575 presented high accuracy, sensitivity, and accuracy in differentiating EP from healthy participants with the area under the curves (AUCs) of 0.8066 and 0.8177, respectively (cut-off value = 1.4964 and 1.5298). Moreover, the AUCs of miR-378 and miR-575 in distinguishing Engel I-II EP from healthy controls were 0.7336 and 0.7949, respectively. Additionally, the AUCs of miR-378 and miR-575 in distinguishing Engel III-IV EP from healthy controls were 0.9345 and 0.8933, with the cut-off value of 1.9696 and 1.6184. Collectively, these results suggested that miR-378 and miR-575 were implicated in epileptogenesis and monitoring EP occurrence.

### Dynamic levels of miR-378 and miR-575 before and after surgical treatment

**Figure 4:**
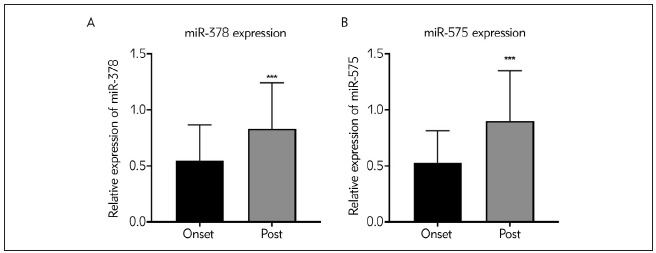
Dynamic expressions of miR-378 and miR-575 in EP patients before and after surgical treatment (A) miR-378 expressions at seizure onset and post-seizure. (B) miR-575 expressions at seizure onset and post-seizure. ***P<0.001, Post vs. Onset. Post, post treatment; onset, epilepsy onset

After surgical treatment, the dynamic changes of miR-378 and miR-575 in EP samples were detected using qRT-PCR assay. As shown in Figure 4A and Figure 4B, the expression of miR-378 and miR-575 was augmented after treatment compared to those at seizure onset, implying that miR-378 and miR-575 may function as feasible biomarkers for EP treatment response (^***^P<0.001).

### Surgical outcomes regarding miR-378 and miR-575 in EP

**Figure 5:**
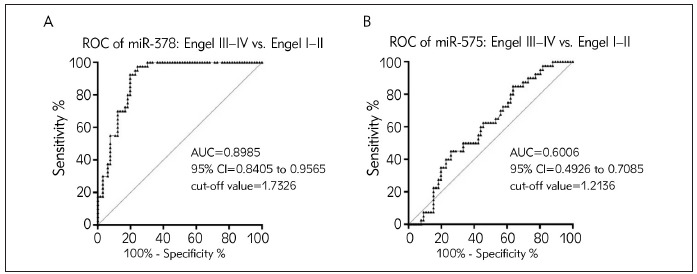
Potentials of aberrant miR-378 and miR-575 regarding surgical outcomes in EP (A) AUC result of miR-378 in Engel I–II epilepsy versus Engel III–IV EP patients. (B) AUC result of miR-575 in Engel I–II epilepsy versus Engel III–IV EP patients

Giving that Engel grade could indicate the surgical outcome in EP, we further examine the AUCs of miR-378 and miR-575 between Engel I-II and Engel III-IV EP patients. As illustrated in Figure 5A, miR-378 could serve as an accurate and high sensitive biomarker for predicting EP surgical prognosis, with an AUC of 0.8985 and a cut-off value of 1.7326. On the contrary, miR-575 had no value in predicting EP surgery outcome with the AUC value of less than 0.8 (Figure 5B).

## Discussion

Statistical reports showed that about 70 million people worldwide were affected by EP [19]. In China, there were more than 9 million EP patients [20]. The number of patients with refractory EP who have been treated regularly for more than 2 years was greater than 2 million, and the prevalence rate accounted for 7‰ of the world's population [21]. The clinical manifestations of epilepsy were diverse, and the typology was complex with 4 common features, seizure, repetitive, transient, and stereotyped [22]. Current treatments for EP included medication, surgery, dietary modifications, and gene therapy [23]. Clinical studies have shown that 70%-80% of new EP patients can be controlled with anti-epileptic drugs (AEDs), and 60%–70% of these patients can be discontinued after 2 to 5 years of treatment with AEDs [24]. Some postoperative patients with EP still needed AEDs to control their symptoms.

Epileptogenesis is a process in which the inflammatory response, the formation of new synaptic and abnormal conduction pathways, and neuronal apop-188 Li et al.: mirR-378 and miR-575 as serum biomarkers of human epilepsytosis were jointly involved [25] [26]. Current studies have shown that a variety of miRNAs are involved in the development of EP. MiR-132 was the first identified miRNA in EP, and its increase led to the development of EP [27]. Furthermore, it was found that miR-34a, miR-132, miR-146a, and miR-184 miRNA expression were upregulated after the seizure, further promoting the recurrence of EP [28]. Meanwhile, miR-124 is decreased in EP, inhibiting seizure activity through targeting CREB1 [29]. Our study found that miR-378 and miR-575 were significantly reduced in serum samples from EP patients in relation to those in healthy controls, which was consistent with a previous study. Similar to our study, another study reported that miR-378 serves as a prognostic biomarker in cholangiocarcinoma [30].

Meanwhile, the clinical utility of microRNA-378 as an early diagnostic biomarker of human cancers has also been documented [31]. Furthermore, miR-378 in serum are potential biomarkers for renal cell carcinoma [32]. Additionally, it has been found that miR-378 play a role in metabolism, angiogenesis, and muscle biology [33]. Similarly, miR-575 serve as a diagnostic marker for metastatic breast cancer and pancreatic cancer [34] [35].

Meanwhile, the ROC analysis validated that miR-378 and miR-575 may be highly sensitive and non-invasive candidates for EP diagnosis. More importantly, according to the Engel guidelines and dynamic changes in miR-378 and miR-575 before and after surgery treatment, we confirmed the potentials of miR-378 and miR-575 in predicting surgery outcome and treatment response in EP as well.

To sum up, miR-378 and miR-575 were decreased in EP serum samples, functioning as feasible biomarkers for EP monitoring and predicting surgical prognosis.

## Dodatak

### Conflict of interest statement

The authors reported no conflict of interest regarding the publication of this article.

## References

[1] Beghi E, Giussani G, Sander J W (2015). The Natural History and Prognosis of Epilepsy. Epileptic Disord.

[2] Boison D, Steinhäuser C (2018). Epilepsy and Astrocyte Energy Metabolism. Glia.

[3] Cheng Y, Mai Q, Zeng X, et al (2019). Propionate Relieves Pentylenetetrazol-Induced Seizures, Consequent Mitochondrial Disruption, Neuron Necrosis and Neurological Deficits in Mice. Biochem Pharmacol.

[4] Li W, Hao N, Xiao Y, Zhou D (2019). Clinical Characteristics and Pregnancy Outcomes of New Onset Epilepsy During Pregnancy. Medicine (Baltimore).

[5] Symonds J D, Zuberi S M, Stewart K, et al (2019). Incidence and Phenotypes of Childhood-Onset Genetic Epilepsies: A Prospective Population-Based National Cohort. Brain.

[6] Zijlmans M, Zweiphenning W, van Klink N (2019). Changing Concepts in Presurgical Assessment for Epilepsy Surgery. Nat Rev Neurol.

[7] Supriya S, Siuly S, Wang H, Zhang Y (2021). Epilepsy Detection from EEG using Complex Network Techniques: A Review. IEEE Rev Biomed Eng.

[8] Ghafouri-Fard S, Shoorei H, Taheri M, Sanak M (2020). Emerging Role of Non-Coding RNAs in Allergic Disorders. Biomed Pharmacother.

[9] Jonas S, Izaurralde E (2015). Towards a Molecular Understanding of MicroRNA-Mediated Gene Silencing. Nat Rev Genet.

[10] Wang J, Zhang C, Peng X, et al (2020). A Combination of Four Serum miRNAs for Screening of Lung Adenocarcinoma. Hum Cell.

[11] Condrat C E, Thompson D C, Barbu M G, et al (2020). miRNAs as Biomarkers in Disease: Latest Findings Regarding Their Role in Diagnosis and Prognosis. Cells.

[12] Ma Y (2018). The Challenge of microRNA as a Biomarker of Epilepsy. Curr Neuropharmacol.

[13] Wang J, Yu J T, Tan L, et al (2015). Genome-Wide Circulating MicroRNA Expression Profiling Indicates Biomarkers for Epilepsy. Sci Rep.

[14] Huang W S, Zhu L (2018). MiR-134 Expression and Changes in Inflammatory Cytokines of Rats with Epileptic Seizures. Eur Rev Med Pharmacol Sci.

[15] Zhang X, Li X, Li B, Sun C, Zhang P (2020). miR-21-5p Protects Hippocampal Neurons of Epileptic Rats Via Inhibiting STAT3 Expression. Adv Clin Exp Med.

[16] Cattani A A, Allene C, Seifert V C, et al (2016). Involvement of MicroRNAs in Epileptogenesis. Epilepsia.

[17] Henshall D C, Hamer H M, Pasterkamp R J, et al (2016). MicroRNAs in Epilepsy: Pathophysiology and Clinical Utility. Lancet Neurol.

[18] Sun J, Cheng W, Liu L, et al (2016). Identification of Serum miRNAs Differentially Expressed in Human Epilepsy at Seizure Onset and Post-Seizure. Mol Med Rep.

[19] Thijs R D, Surges R, O'Brien T J, Sander J W (2019). Epilepsy in Adults. Lancet.

[20] Song P, Liu Y, Yu X, et al (2017). Prevalence of Epilepsy in China Between 1990 and 2015: A Systematic Review and Meta-analysis. J Glob Health.

[21] Zhao R, Xue P, Zhou Y, et al (2020). Application of Robot-Assisted Frameless Stereoelectroencephalography Based on Multimodal Image Guidance in Pediatric Refractory Epilepsy: Experience of a Pediatric Center in a Developing Country. World Neurosurg.

[22] Padmanaban V, Inati S, Ksendzovsky A, Zaghloul K (2019). Clinical Advances in Photosensitive Epilepsy. Brain Res.

[23] Wijnen B F M, van Mastrigt G, Evers S, et al (2017). A Systematic Review of Economic Evaluations of Treatments for Patients with Epilepsy. Epilepsia.

[24] Nolan S J, Tudur Smith C, Weston J, Marson A G (2016). Lamotrigine Versus Carbamazepine Monotherapy for Epilepsy: An Individual Participant Data Review. Cochrane Libr.

[25] Wei H, Xu Y, Chen Q, et al (2020). Mesenchymal Stem Cell-Derived Exosomal miR-223 Regulates Neuronal Cell Apoptosis. Cell Death Dis.

[26] Ye Y, Xu H, Su X, He X (2016). Role of MicroRNA in Governing Synaptic Plasticity. Neural Plast.

[27] Wang X, Zhou Y, Gao Q, et al (2020). The Role of Exosomal microRNAs and Oxidative Stress in Neurodegenerative Diseases. Oxid Med Cell Longev.

[28] Galardi A, Colletti M, Businaro P, et al (2018). MicroRNAs in Neuroblastoma: Biomarkers with Therapeutic Potential. Curr Med Chem.

[29] Sueri C, Gasparini S, Balestrini S, et al (2018). Diagnostic Biomarkers of Epilepsy. Curr Pharm Biotechnol.

[30] Zhou Z, Ma J (2019). miR-378 Serves as a Prognostic Biomarker in Cholangiocarcinoma and Promotes Tumor Proliferation, Migration, and Invasion. Cancer Biomark.

[31] Li Z, Shen L, Li Y, et al (2016). Clinical Utility of MicroRNA-378 as Early Diagnostic Biomarker of Human Cancers: A Meta-Analysis of Diagnostic Test. Oncotarget.

[32] Redova M, Poprach A, Nekvindova J, et al (2012). Circulating miR-378 and miR-451 in Serum Are Potential Biomarkers for Renal Cell Carcinoma. J Transl Med.

[33] Krist B, Florczyk U, Pietraszek-Gremplewicz K, et al (2015). The Role of miR-378a in Metabolism, Angiogenesis, and Muscle Biology. Int J Endocrinol.

[34] Satomi-Tsushita N, Shimomura A, Matsuzaki J, et al (2019). Serum MicroRNA-Based Prediction of Responsiveness to Eribulin in Metastatic Breast Cancer. PLoS One.

[35] Yan Q, Hu D, Li M, et al (2020). The Serum MicroRNA Signatures for Pancreatic Cancer Detection and Operability Evaluation. Front Bioeng Biotechnol.

